# Efficacy and Safety of Viltolarsen in Boys With Duchenne Muscular Dystrophy: Results From the Phase 2, Open-Label, 4-Year Extension Study

**DOI:** 10.3233/JND-221656

**Published:** 2023-05-02

**Authors:** Paula R. Clemens, Vamshi K. Rao, Anne M. Connolly, Amy D. Harper, Jean K. Mah, Craig M. McDonald, Edward C. Smith, Craig M. Zaidman, Tomoyuki Nakagawa, the CINRG DNHS Investigators, Eric P. Hoffman

**Affiliations:** a Department of Neurology, University of Pittsburgh School of Medicine, Pittsburgh, PA, USA; b Department of Veterans Affairs Medical Center, Pittsburgh, PA, USA; c Division of Neurology, Ann and Robert H. Lurie Children’s Hospital of Chicago, Chicago, IL, USA; d Division of Neurology, Nationwide Children’s Hospital, The Ohio State University College of Medicine, Columbus, OH, USA; e Children’s Hospital of Richmond at Virginia Common wealth University, Richmond, VA, USA; f Department of Pediatrics, University of Calgary, Calgary, Alberta, Canada; g Department of Physical Medicine and Rehabilitation, Department of Pediatrics, UC Davis Health, University of California, Davis, Sacramento, CA, USA; h Department of Pediatrics, Duke University Medical Center, Durham, NC, USA; i Department of Neurology, Washington University at St Louis, St Louis, MO, USA; j NS Pharma, Inc., Paramus, NJ, USA; k See Supplemental Table 1; l Department of Pharmaceutical Sciences, Binghamton University – State University of New York, Binghamton, NY

**Keywords:** Duchenne muscular dystrophy, dystrophin, viltolarsen, exon skipping, clinical efficacy

## Abstract

**Background:**

Duchenne muscular dystrophy (DMD) is caused by DMD gene mutations, resulting in absence of functional dystrophin protein. Viltolarsen, an exon 53 skipping therapy, significantly increased dystrophin levels in patients with DMD. Presented here are completed study results of > 4 years of functional outcomes in viltolarsen-treated patients compared to a historical control group (Cooperative International Neuromuscular Research Group Duchenne Natural History Study [CINRG DNHS]).

**Objective:**

To evaluate the efficacy and safety of viltolarsen for an additional 192 weeks in boys with DMD.

**Methods:**

This phase 2, open-label, 192-week long-term extension (LTE) study (NCT03167255) evaluated the efficacy and safety of viltolarsen in participants aged 4 to < 10 years at baseline with DMD amenable to exon 53 skipping. All 16 participants from the initial 24-week study enrolled into this LTE. Timed function tests were compared to the CINRG DNHS group. All participants received glucocorticoid treatment. The primary efficacy outcome was time to stand from supine (TTSTAND). Secondary efficacy outcomes included additional timed function tests. Safety was continuously assessed.

**Results:**

For the primary efficacy outcome (TTSTAND), viltolarsen-treated patients showed stabilization of motor function over the first two years and significant slowing of disease progression over the following two years compared with the CINRG DNHS control group which declined. Viltolarsen was well tolerated, with most reported treatment-emergent adverse events being mild or moderate. No participants discontinued drug during the study.

**Conclusions:**

Based on the results of this 4-year LTE, viltolarsen can be an important treatment strategy for DMD patients amenable to exon 53 skipping.

## INTRODUCTION

Duchenne muscular dystrophy (DMD) is an X-linked, recessive, neuromuscular disorder resulting from DMD gene mutations (deletions, duplications, nonsense mutations) causing a loss of dystrophin protein and function in striated muscle [1, 2]. DMD occurs in approximately 1 : 3600 to 1 : 9300 male births [1]. Patients with DMD experience degeneration of skeletal muscle and resultant muscle weakness, resulting in respiratory failure and cardiac insufficiency leading to premature death [3–5]. However, new therapeutic advances for DMD have emerged that include exon skipping and gene therapy, with a goal to slow disease progression [5–8]. More specifically, exon skipping therapies designed for the treatment of DMD are capable of providing expression of truncated dystrophin protein in skeletal muscle and, in return, may delay the progression of the disease [9, 10].

Exon skipping therapies use antisense oligonucleotides to restore the open reading frame of the pre-messenger RNA by changing DMD out-of-frame deletions to in-frame deletions [11, 12]. Approximately 8% to 10% of patients with DMD have DMD gene deletions that are amenable to exon 53 skipping, a treatment approach that can restore the reading frame and promote production of an internally shortened dystrophin protein, which has the capability to provide partial function [2, 11, 12]. Viltolarsen is an antisense oligonucleotide designed to treat DMD in patients with a confirmed mutation of the DMD gene amenable to exon 53 skipping [13]. Viltolarsen is approved in the US by the Food and Drug Administration (FDA) and in Japan for the treatment of DMD based on a significant increase in muscle dystrophin expression that was evident in clinical trials [10, 13–15].

An initial phase 2, randomized, 24-week clinical trial that evaluated the efficacy, safety, and tolerability of viltolarsen in boys 4 to < 10 years of age with DMD demonstrated muscle dystrophin production [13]. All 16 participants showed an increase in dystrophin levels, measured by a validated western blot assay at the end of the 24 weeks of treatment [13]. Further, a mean dystrophin value of 5.9% was achieved at the recommended 80 mg/kg/week dosage [13]. The western blot data were supported by significant increases in dystrophin mRNA splicing on reverse transcription–polymerase chain reaction, dystrophin protein by mass spectrometry, and percentage of dystrophin-positive myofibers by immunohistochemistry [13]. Participants treated with viltolarsen showed improvements in timed function tests from baseline for time to stand from supine (TTSTAND) and time to run/walk 10 meters (TTRW) compared with a group-matched historical control group (Cooperative International Neuromuscular Research Group Duchenne Natural History Study [CINRG DNHS]) [13]. Overall, viltolarsen was well tolerated, with no reports of treatment-related serious adverse events (SAEs), discontinuations, or deaths occurring in the study [13]. At the conclusion of the 24-week study, all participants were given the opportunity to enroll in the long-term extension (LTE) study (NCT03167255) [13].

The primary objective of the LTE study was to evaluate the effects on mobility and safety of 40- or 80-mg/kg/week intravenous doses of viltolarsen for an additional 192-week treatment period for a total of 216 weeks of treatment from the start of the initial phase 2 study to the completion of the open-label extension study. An interim analysis at 2 years was published, and the data presented here are the final results following 4 years of treatment [10].

## MATERIALS AND METHODS

### Participants and trial design

**Fig. 1:**
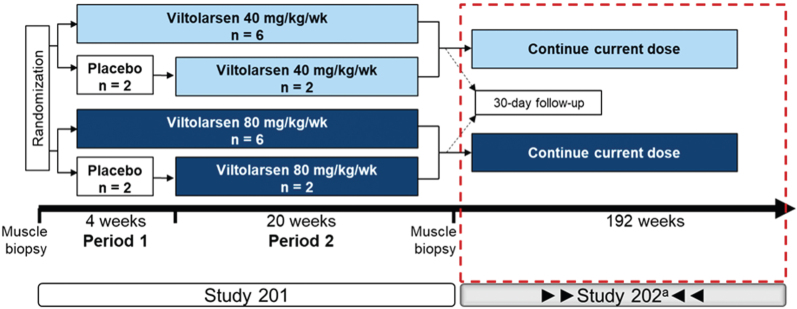
Study design. ^a^The first participant who received 40 mg/kg/week was increased to the higher dose (80 mg/kg/week) at wk 178 with the remaining participants switching to the higher dose by wk 197. Wk, week.

All participants (*N* = 16) of the initial 24-week, phase 2 study (NCT02740972) continued into the phase 2, open-label, LTE study to assess long-term efficacy, assessed by timed muscle function tests, and safety of viltolarsen for up to an additional 192 weeks (NCT03167255; Fig. 1). Participant enrollment occurred at six sites across the US and Canada. Participants (4 to < 10 years of age when enrolled in the initial phase 2 study) continued to receive weekly viltolarsen at the dosage received in the initial 24-week study. Participants enrolled in the LTE study received treatment for the duration of the study (up to 216 weeks of total treatment, including initial study, plus a 30-day posttreatment phase). Viltolarsen was administered as an intravenous infusion over one hour at a dosage of 40 or 80 mg/kg once weekly. Following the Institutional Review Board approval (and per advisement from the US FDA), all participants who were receiving 40 mg/kg/week since the start of the initial study were dose increased to 80 mg/kg/week for the remainder of their participation in the LTE study. The first participant in the low-dose cohort was switched at week 178 and the remaining seven participants were switched by week 197. Participants were required to remain on a stable dose of glucocorticoid for the duration of the study. Although no participants were excluded, exclusion criteria included all those who experienced SAEs or severe AEs in the initial study that were related to the study drug, and those who received treatment for dystrophin or dystrophin-related protein indication or other new investigational drugs after completion of the initial 24-week study. Populations representing gene deletions amenable to exon skipping of exon 44 or deletions of exons 1–8 were not represented as a comparator in the historical control group because these mutation groups typically have a milder disease progression. The clinical study protocol was approved by the Institutional Review Board and this study was performed according to the Good Clinical Practice and International Conference on Harmonization regulations, the code of federal regulations, US FDA, and principles of the Declaration of Helsinki.

### Outcomes and assessments

Efficacy assessments were performed every 12 weeks. Efficacy was measured by timed function tests, including TTSTAND (primary endpoint), secondary endpoints of TTRW, time to climb 4 stairs (TTCLIMB), North Star Ambulatory Assessment (NSAA), and six-minute walk test (6MWT). The timed results for TTSTAND, TTCLIMB, and TTRW were converted to velocities, and both time and velocity results are presented. The rationale for converting to velocity is to provide a way to include participants who were no longer able to perform the tests mentioned in the analysis for that visit. Efficacy assessments were compared to historical controls (CINRG DNHS), who were group matched for geographic location, age, glucocorticoid use, and ambulatory ability at baseline, defined as all participants being able to execute TTSTAND, TTRW, and TTCLIMB, at baseline. The last time point in which efficacy assessments were available for both the viltolarsen and CINRG DNHS groups was week 205 due to the schedule of CINRG DNHS assessments; therefore, week 205 is the last assessment date in the analysis set. Safety was assessed at each visit or participant contact throughout the duration of the study. Treatment-emergent adverse events (TEAEs) and SAEs were assessed during the safety analysis. Before performing any study-related activities, parents or legal guardians of the participants provided written informed consent and Health Insurance Portability and Accountability Act authorization.

### Statistical analysis

The sample size in this study was determined from the participants who completed the initial 24-week study and enrolled into the LTE study. Therefore, the sample size was not based on any statistical considerations. Statistical considerations and power to detect statistical differences were aligned with the initial 24-week study [13].

All timed function tests were performed using the full analysis set (FAS), which included all participants who received≥1 dose of viltolarsen in both the initial 24-week study and LTE study. The safety population, which was the primary analysis population for safety assessments, was identical to the FAS. Efficacy outcome measures were tested between study participants and the CINRG DNHS control group using a mixed-effects linear model. All statistical tests were two-sided and performed at a significance level of 0.05 using SAS v9.4 or higher.

## RESULTS

### Participants

All participants (N = 16) who completed the initial 24-week, phase 2 study qualified and transitioned into the LTE study. The majority of participants were White (15/16, 94%), with mean age in the LTE study being 7.9 years. Two participants enrolled when they were 4 years old. Overall, baseline characteristics between participants in the two dosage cohorts were balanced and similar to the CINRG DNHS controls (Table 1). All participants in the study and the CINRG DNHS external control set received chronic treatment with glucocorticoids.

**Table 1:** Participant baseline demographics

	Viltolarsen cohort, mean			CINRG DNHS control cohort, mean		
Characteristics	40 mg/kg/wk	80 mg/kg/wk	Total	Exon 53 amenable	Non-exon 53 amenable	Total
	(*n* = 8)	(*n* = 8)	(*N* = 16)	controls (*n* = 9)	controls (*n* = 56)	(*N* = 65)
Age, years	7.5	7.2	7.4	6.3	7.2	7.1
Weight, kg	23.7	22.3	23.0	21.6	24.4	24.0
Height, cm	114.6	112.2	113.4	111.3	116.6	115.8
BMI, kg/m^2^	17.9	17.4	17.6	17.3	17.5	17.5

BMI, body mass index; CINRG, Cooperative International Neuromuscular Research Group; DNHS, Duchenne Natural History Study.

### Efficacy outcomes

**Fig. 2:**
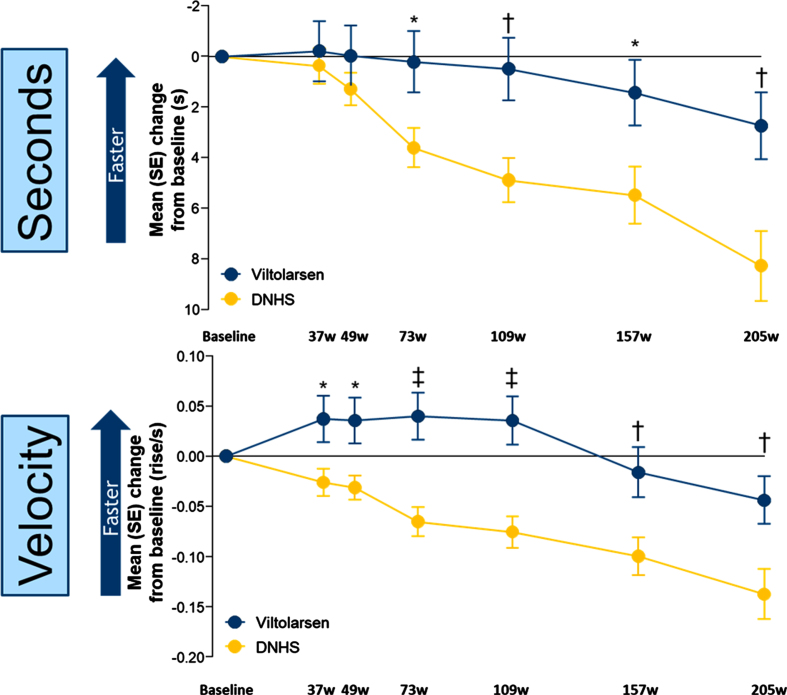
Timed Function Tests: Change From Baseline vs Natural History Controls for TTSTAND. ^*^P<0.05; ^†^*P*<0.01; ^‡^*P*≤0.001. DNHS, Duchenne Natural History Study; s, seconds; SE, standard error; TTSTAND, time to stand from supine; w, weeks. TTSTAND (seconds) Viltolarsen sample size (n): 16, 16, 15, 14, 14, 11, 13. TTSTAND (seconds) CINRG DNHS sample size (n): 65, 31, 57, 24, 24, 14, 10. TTSTAND (velocity) Viltolarsen sample size (n): 16, 16, 15, 14, 14, 11, 14. TTSTAND (velocity) CINRG DNHS sample size (n): 65, 31, 58, 28, 28, 20, 12.

Timed function tests were used to indicate DMD progression. For the primary efficacy endpoint (TTSTAND) participants who received viltolarsen showed stabilization of motor function over the first two years and a significant slowing of motor function loss over the following two years, whereas a more significant decline was observed in the CINRG DNHS comparator group over the entire 4-year period (Fig. 2). Change from baseline improvements were statistically significant (*P* < 0.05) for TTSTAND (seconds) beginning at week 73 and remained significantly different through week 205 (Fig. 2A), and for TTSTAND (velocity) beginning at week 37 and remained significantly (*P* < 0.05) different through week 205 (Fig. 2B).

**Fig. 3:**
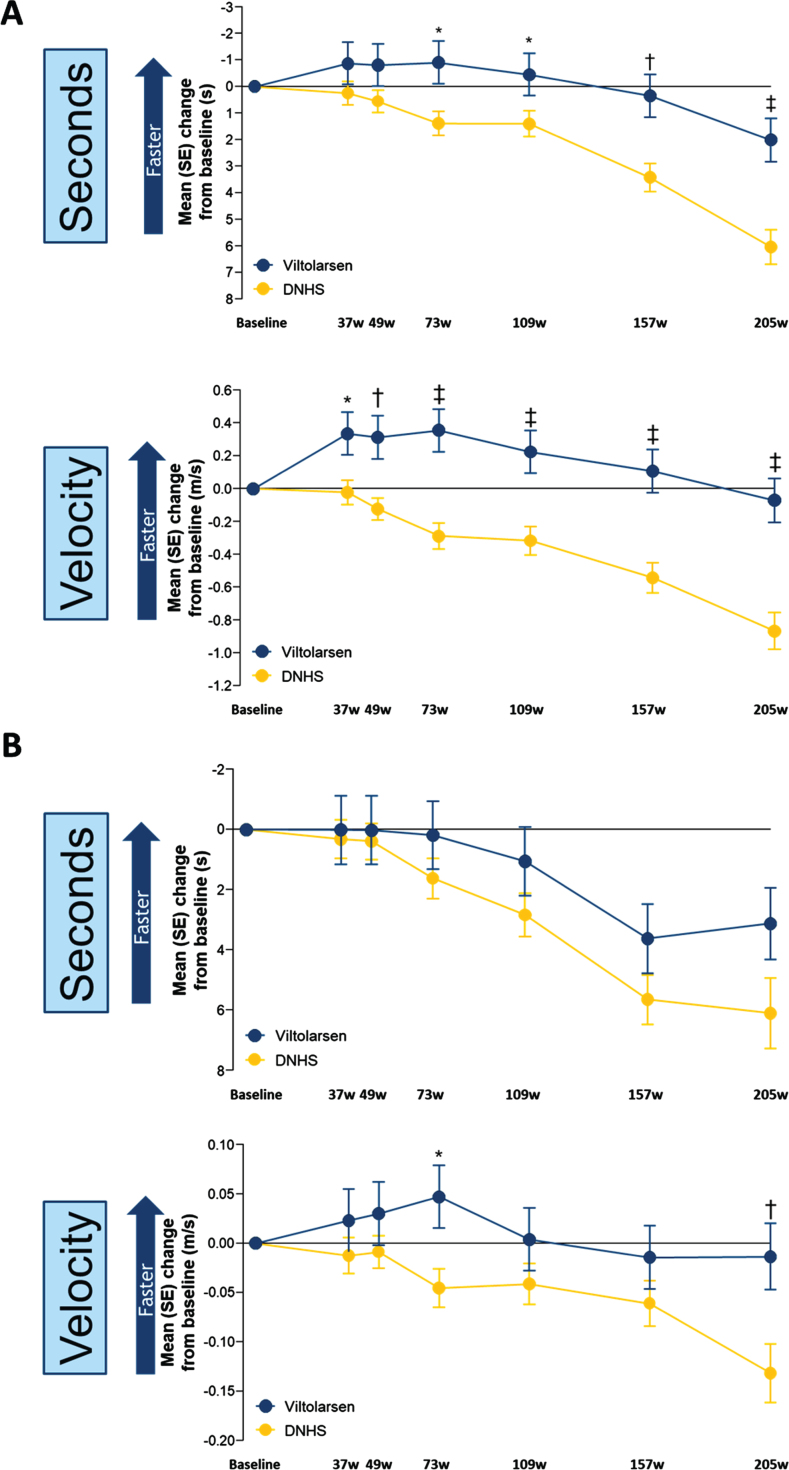
Timed Function Tests: Change From Baseline vs Natural History for TTRW and TTCLIMB. A. TTRW change from baseline vs natural history controls. B. TTCLIMB change from baseline vs natural history controls. ^*^P < 0.05; ^†^*P* < 0.01; ^‡^*P*≤0.001. DNHS, Duchenne Natural History Study; s, seconds; SE, standard error; TTCLIMB, time to climb 4 stairs; TTRW, time to run/walk 10 meters; w, weeks. TTRW (seconds) Viltolarsen sample size (n): 16, 16, 15, 16, 16, 14, 14. TTRW (seconds) CINRG DNHS sample size (n): 65, 32, 58, 28, 29, 26, 16. TTRW (velocity) Viltolarsen sample size (n): 16, 16, 15, 16, 16, 14, 14. TTRW (velocity) CINRG DNHS sample size (n): 65, 32, 59, 29, 29, 27, 19. TTCLIMB (seconds) Viltolarsen sample size (n): 16, 16, 14, 16, 16, 14, 13. TTCLIMB (seconds) CINRG DNHS sample size (n): 65, 33, 56, 28, 30, 23, 10. TTCLIMB (velocity) Viltolarsen sample size (n): 16, 16, 14, 16, 16, 14, 13. TTCLIMB (velocity) CINRG DNHS sample size (n): 65, 33, 57, 29, 30, 26, 16.

Similarly, the change from baseline for TTRW showed stabilization of motor function over the first two years and significant slowing of motor function loss over the following two years for viltolarsen-treated participants compared with the CINRG DNHS comparator group (Fig. 3A). The change from baseline (seconds) was significant for TTRW at the beginning of week 73 (*P* = 0.01) and remained significantly different through week 205 (*P*≤0.0001) and for TTRW (velocity) beginning at week 37 (*P* = 0.01) and remained significantly different through week 205 (*P*≤0.0001). TTCLIMB (seconds) did not show a significant difference between the viltolarsen and the CINRG DNHS comparator group, whereas TTCLIMB (velocity) was significant at week 73 (*P* = 0.01) and week 205 (*P* = 0.007) (Fig. 3B). 6MWT and NSAA efficacy endpoints were added later in the clinical study to the CINRG DNHS protocol, and as a result the historical comparator control group did not have sufficient data on 6MWT and NSAA to adequately compare with the viltolarsen-treated participants [10]. The 6MWT distances over the 4-year period for the viltolarsen-treated group similarly showed stable measures, as did NSAA measures (Supplementary Fig. 1).

### Safety

The safety profile of viltolarsen over the course of the LTE study was acceptable and comparable to that observed in the initial 24-week study. TEAEs were reported by all 16 participants and were primarily categorized as mild or moderate in severity (Table 2). Only one mild TEAE of injection site extravasation (80 mg/kg/week treatment group) was assessed as related to the study drug, which resolved the same day. The most frequently reported TEAEs by preferred term (≥25% of participants) in the LTE study overall were cough, nasopharyngitis, fall, influenza, insect bite, contusion, nasal congestion, vomiting, headache, pyrexia, and rash (Table 2). There were four SAEs reported in three participants and all were categorized by the investigators as unrelated to study medication, including left tibia and fibula fracture, right femur fracture, rhabdomyolysis, and left tibia and fibula fracture. No action was taken with the study drug, and all of these SAEs resolved with patients having recovered. Laboratory data on renal function (including cystatin C) were followed for the whole length of the study, with no clinically significant findings being reported. No participants discontinued the study drug due to treatment-emergent SAEs or AEs, and no deaths occurred during the study.

**Table 2:** Safety profile of viltolarsen and common TEAEs (Preferred Term in≥25% of Participants)

Participants with	Viltolarsen treatment		Total
	40 mg/kg/wk	80 mg/kg/wk
	(*n* = 8)	(*n* = 8)	(*N* = 16)
Any TEAE, *n* (%)	8 (100)	8 (100)	16 (100)
Any drug-related TEAE, *n* (%)	0	1 (13)^a^	1 (6)
Any serious TEAE, *n* (%)	1 (13)	2 (25)	3 (19)
Study drug discontinuation due to TEAE, *n* (%)	0	0	0
Death, *n* (%)	0	0	0
AE, *n* (%)
Cough	5 (63)	5 (63)	10 (63)
Nasopharyngitis	4 (50)	5 (63)	9 (56)
Insect bite	4 (50)	2 (25)	6 (38)
Rash	2 (25)	4 (50)	6 (38)
Vomiting	3 (38)	3 (38)	6 (38)
Fever	2 (25)	3 (38)	5 (31)
Fall	4 (50)	1 (13)	5 (31)
Headache	3 (38)	2 (25)	5 (31)
Nasal congestion	3 (38)	2 (25)	5 (31)
Influenza	3 (38)	1 (13)	4 (25)
Diarrhea	1 (13)	2 (25)	3 (19)

^a^Assessed as injection site extravasation. AE, adverse event; TEAE, treatment-emergent AE; wk, week.

## DISCUSSION

This was an open-label LTE study that assessed the efficacy and safety of viltolarsen for 192 weeks in boys with DMD. The results of timed function tests, such as TTSTAND, TTRW, and TTCLIMB, are key indicators for assessing the progression of DMD [16]. These data, combined with the initial 24-week study, provide over 4 years of functional outcomes data—the longest such study and the only one with positive, significant motor function outcomes for an exon 53 skipping therapy [17]. The observed outcomes of maintained clinical performance in timed function tests over the first two years, followed by a significant slowing of disease progression over the next two years, was demonstrated in the viltolarsen-treated participants, whereas the prospectively collected comparator control group showed a more significant functional decline over the entire course of the 4-year study. The differences in reported motor outcomes between viltolarsen and golodirsen, another exon 53 skipping agent with long-term data available, may be associated with the extent of dystrophin rescue, as viltolarsen has shown a mean dystrophin increase in muscle of 6%, whereas golodirsen administration over 48 weeks resulted in dystrophin protein being present at  1% [13, 17].

DMD is a progressive disease with current therapies aiming to delay motor decline for as long as possible, providing meaningful improvement in quality of life. The TTSTAND and TTRW timed function tests in the viltolarsen treatment group showed clear and significant differences over the 4-year study period compared with the CINRG DNHS group, which was matched for key factors, including age, baseline ambulatory ability, steroid use, and geographic location. The TTCLIMB assessment in the viltolarsen treatment group was numerically better (seconds) and significantly better (velocity, weeks 37 and 205) than the CINRG DNHS group, but the overall effect was less pronounced. There may be several reasons for this observation. First, there is greater variability in the TTCLIMB data compared to TTSTAND and TTRW. This may be due, in part, to some boys using a compensation strategy during the TTCLIMB test (eg, using the available handrail to complete the task). Second, fatigue could be a factor since TTCLIMB was not the first test administered. Interestingly, when the seconds measured was transformed to velocity, the viltolarsen-treated group had an essentially flat and stable profile throughout the 205 weeks, whereas the CINRG DNHS group still showed decline, thus indicating a viltolarsen treatment effect.

Viltolarsen was well tolerated in this study, with most of the reported TEAEs being mild or moderate with no deaths or study discontinuations. No treatment-related SAEs and no new or unexpected safety findings with viltolarsen were observed in this LTE study. Most adverse events were consistent with conditions expected in a pediatric population with DMD.

### Limitations

Limitations of this study include the small number of participants and the lack of a placebo control arm. With DMD occurring in only approximately 1 : 3600 to 1 : 9300 male births, and only 8% –10% of patients having DMD who are amenable to exon 53 skipping, [1, 2] there are clear challenges in recruiting large numbers of patients. The small sample size (*N* = 16) in this study is consistent with other studies investigating treatment options for this patient population [13, 17, 18]. The use of a historical group over a placebo arm is less rigorous than a randomized, placebo-controlled study design. However, the CINRG DNHS control group was matched to the viltolarsen group on key criteria, including age, ambulatory ability, glucocorticoid treatment, and geographic location. Additionally, deletion of exons 1–8 and patients amenable to exon 44 skipping, which have milder disease progression, were excluded from the historical control group, allowing for a better match between participants in the LTE study and the CINRG DNHS control group. Finally, it is important to acknowledge that the best comparators in this study are participants with DMD amenable to exon 53 skipping (*n* = 9 in this study). However, recent evidence from the Collaborative Trajectory Analysis Project showed that participant genotypes in DMD studies have a limited effect on motor outcomes, suggesting the viability of trial designs that incorporate genotypically mixed or unmatched controls, supporting the use of mixed genotypes in the control group in this study [19].

## Conclusions

The study outcome of improved motor function vs historical controls, and a favorable safety profile, have been demonstrated in the longest exon 53 skipping therapy trial to date. This is the only study of an exon 53 skipping agent used to demonstrate significant functional benefit over 4 years with comparison to a group-matched, prospectively collected, control group. Based on the efficacy and safety data reported here, combined with the previously reported significant increase in dystrophin levels in these same participants, viltolarsen can be an important part of the treatment strategy for DMD patients who have mutations that are amenable to exon 53 skipping.

## Supplementary Material

Supplementary Material
