# Supplementary material for: Efficacy and Safety of Viltolarsen in Boys With Duchenne Muscular Dystrophy: Results From the Phase 2, Open-Label, 4-Year Extension Study
Source: J Neuromuscul Dis. 2023 May 2;10(3):439–47. doi: 10.3233/JND-221656 (PMC10200237; doi:10.3233/JND-221656)
Supplement: Supplementary Material [file jnd-10-jnd221656-s001.docx]

**SUPPLEMENTARY MATERIAL**

**Supplementary Table 1.** CINRG DNHS Investigators and Affiliations

| **University of California, Davis, Sacramento, California, USA** |
| --- |
| C.M. McDonald |
| E.K. Henricson |
| R.T. Abresch |
| N.C. Joyce |
| **Sundaram Medical Foundation and Apollo Children’s Hospital, Chennai, India** |
| V. Vishwanathan |
| S. Chidambaranathan |
| **Holland Bloorview Kids Rehab Hospital, Toronto, Ontario, Canada** |
| W.D. Biggar |
| L.C. McAdam |
| **Alberta Children’s Hospital, Calgary, Alberta, Canada** |
| J.K. Mah |
| **Queen Silvia Children’s Hospital, Göteborg, Sweden** |
| M. Tulinius |
| **Children’s National Medical Center, Washington DC, USA** |
| A. Cnaan |
| L.P. Morgenroth |
| R. Leshner |
| C. Tesi-Rocha |
| M. Thangarajh |
| T. Duong |
| **Royal Children’s Hospital, Melbourne, Victoria, Australia** |
| A. Kornberg |
| M. Ryan |
| **Hadassah Hebrew University Hospital, Jerusalem, Israel** |
| Y. Nevo |
| **Instituto de Neurociencias Fundacion Favaloro, Buenos Aires, Argentina** |
| A. Dubrovsky |
| **University of Pittsburgh and Children’s Hospital of Pittsburgh, Pittsburgh, Pennsylvania, USA** |
| P.R. Clemens |
| H. Abdel-Hamid |
| **Washington University in St Louis, St Louis, Missouri, USA** |
| A.M. Connolly |
| A. Pestronk |
| **Children’s Hospital of Virginia, Richmond, Virginia, USA** |
| J. Teasley |
| **University of Tennessee, Memphis, Tennessee, USA** |
| T.E. Bertorini |
| **Children’s Hospital at Westmead, Sydney, New South Wales, Australia** |
| R. Webster |
| **University of Alberta, Edmonton, Alberta, Canada** |
| H. Kolski |
| **Mayo Clinic, Rochester, Minnesota, USA** |
| N. Kuntz |
| S. Driscoll |
| J.B. Bodensteiner |
| **University of Puerto Rico, San Juan, Puerto Rico** |
| J. Carlo |
| **University of Pavia and Niguarda Ca’ Granda Hospital, Milan, Italy** |
| K. Gorni |
| **Texas Children’s Hospital, Houston, Texas, USA** |
| T. Lotze |
| **University of Minnesota, Minneapolis, Minnesota, USA** |
| J.W. Day |
| P. Karachunski |

**Supplementary Fig. 1.** Six-Minute Walk Distances and North Star Ambulatory Assessment for Viltolarsen-Treated Participants Over 4 years of treatment

**
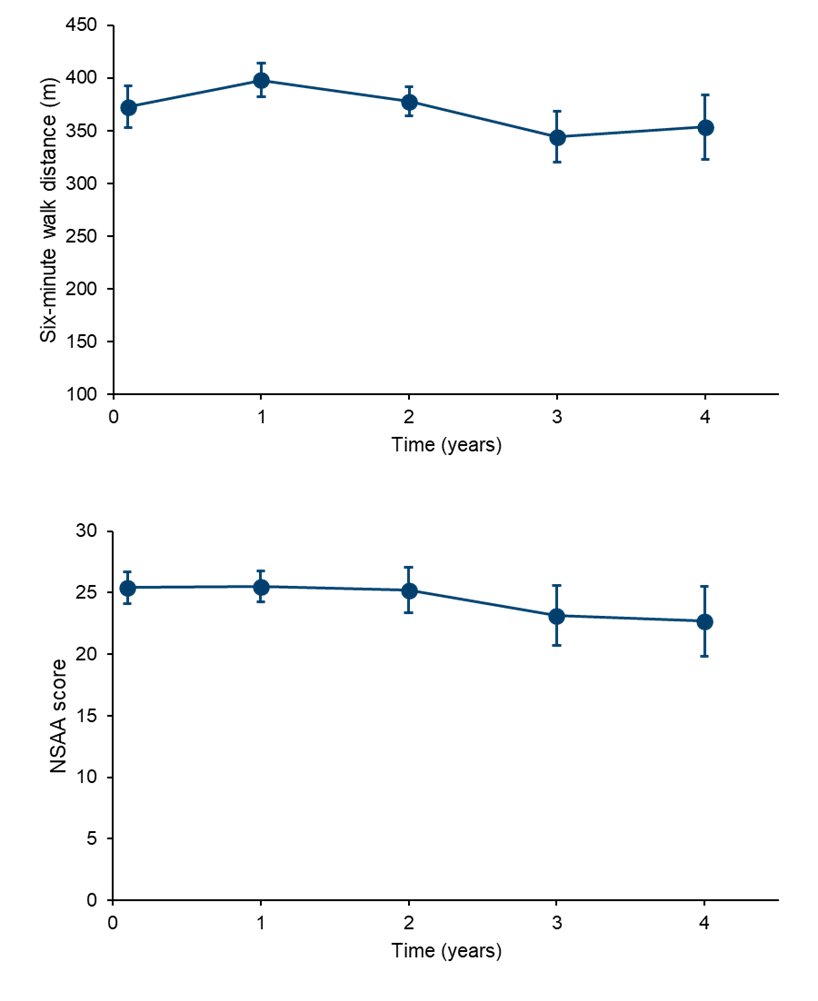
**

Six-minute walk distance sample size (n): 16, 14, 16, 13, 14

NSAA score sample size (n): 16, 15, 16, 14, 14

NSAA, North Star Ambulatory Assessment.
